# Effectiveness of a Web-Based Brief Alcohol Intervention and Added Value of Normative Feedback in Reducing Underage Drinking: A Randomized Controlled Trial

**DOI:** 10.2196/jmir.1465

**Published:** 2010-12-19

**Authors:** Renske Spijkerman, Marion AE Roek, Ad Vermulst, Lex Lemmers, Annemarie Huiberts, Rutger CME Engels

**Affiliations:** ^2^Trimbos InstituteUtrechtNetherlands; ^1^Behavioural Science InstituteDepartment of Developmental PsychopathologyRadboud UniversityNijmegenNetherlands

**Keywords:** Web-based brief alcohol intervention, adolescents, normative feedback, moderate drinking, alcohol use

## Abstract

**Background:**

Current insights indicate that Web-based delivery may enhance the implementation of brief alcohol interventions. Previous research showed that electronically delivered brief alcohol interventions decreased alcohol use in college students and adult problem drinkers. To date, no study has investigated the effectiveness of Web-based brief alcohol interventions in reducing alcohol use in younger populations.

**Objective:**

The present study tested 2 main hypotheses, that is, whether an online multicomponent brief alcohol intervention was effective in reducing alcohol use among 15- to 20-year-old binge drinkers and whether inclusion of normative feedback would increase the effectiveness of this intervention. In additional analyses, we examined possible moderation effects of participant’s sex, which we had not a priori hypothesized.

**Method:**

A total of 575 online panel members (aged 15 to 20 years) who were screened as binge drinkers were randomly assigned to (1) a Web-based brief alcohol intervention without normative feedback, (2) a Web-based brief alcohol intervention with normative feedback, or (3) a control group (no intervention). Alcohol use and moderate drinking were assessed at baseline, 1 month, and 3 months after the intervention. Separate analyses were conducted for participants in the original sample (n = 575) and those who completed both posttests (n = 278). Missing values in the original sample were imputed by using the multiple imputation procedure of PASW Statistics 18.

**Results:**

Main effects of the intervention were found only in the multiple imputed dataset for the original sample suggesting that the intervention without normative feedback reduced weekly drinking in the total group both 1 and 3 months after the intervention (n =575, at the 1-month follow-up, beta = -.24, *P =* .05; at the 3-month follow-up, beta = -.25, *P =* .04). Furthermore, the intervention with normative feedback reduced weekly drinking only at 1 month after the intervention (n=575, beta = -.24, *P =* .008). There was also a marginally significant trend of the intervention without normative feedback on responsible drinking at the 3-month follow-up (n =575, beta = .40, *P =*.07) implying a small increase in moderate drinking at the 3-month follow-up. Additional analyses on both datasets testing our post hoc hypothesis about a possible differential intervention effect for males and females revealed that this was the case for the impact of the intervention without normative feedback on weekly drinking and moderate drinking at the 1-month follow-up (weekly drinking for n = 278, beta = -.80, *P =* .01, and for n = 575, beta = -.69, *P =* .009; moderate drinking for n = 278, odds ratio [OR] = 3.76, confidence interval [CI] 1.05 - 13.49, *P =* .04, and for n = 575, OR = 3.00, CI = 0.89 - 10.12, *P =* .08) and at the 3-month follow-up (weekly drinking for n = 278, beta = -.58, *P =* .05, and for n = 575, beta = -.75, *P =* .004; moderate drinking for n = 278, OR = 4.34, CI = 1.18 - 15.95, *P =* .04, and for n = 575, OR = 3.65, CI = 1.44 - 9.25, *P =* .006). Furthermore, both datasets showed an interaction effect between the intervention with normative feedback and participant’s sex on weekly alcohol use at the 1-month follow-up (for n = 278, beta = -.74, *P =*.02, and for n = 575, beta = -.64, *P =*.01) and for moderate drinking at the 3-month follow-up (for n = 278, OR = 3.10, CI = 0.81 - 11.85, *P =* .07, and for n = 575, OR = 3.00, CI = 1.23 - 7.27, *P =* .01). Post hoc probing indicated that males who received the intervention showed less weekly drinking and were more likely to drink moderately at 1 month and at 3 months following the intervention. For females, the interventions yielded no effects: the intervention without normative feedback even showed a small unfavorable effect at the 1-month follow-up.

**Conclusion:**

The present study demonstrated that exposure to a Web-based brief alcohol intervention generated a decrease in weekly drinking among 15- to 20-year-old binge drinkers but did not encourage moderate drinking in the total sample. Additional analyses revealed that intervention effects were most prominent in males resulting in less weekly alcohol use and higher levels of moderate drinking among 15- to 20-year-old males over a period of 1 to 3 months.

**Trial Registration:**

ISRCTN50512934; http://www.controlled-trials.com/ISRCTN50512934/ (Archived by WebCite at http://www.webcitation.org/5usICa3Tx)

## Introduction

Numerous studies have indicated that early drinking onset and excessive alcohol use can have detrimental consequences for adolescents’ current and future health status [1-4]. Compared with adults, adolescents appear more susceptible to the harmful effects of alcohol due to biological, psychological, and social developmental changes that typically occur during the adolescent life stage [5,6]. In addition, findings suggest that alcohol consumption can harm the developing adolescent brain, which eventually could lead to deficits in neurocognitive functioning [7,8]. The accumulating insights into alcohol-related health hazards for adolescents have caused great concern among national and international health authorities and have resulted in plans for action to reduce underage and hazardous drinking among youth [9,10]. Recent evidence suggests that these intensified alcohol prevention activities seem to pay off by showing a steady decline in alcohol consumption levels among adolescents in the United States [11] and also in the Netherlands [12]. However, despite these recently reported decreases in alcohol prevalence rates, the proportion of early and heavy drinking adolescents is still considerably high. For example, data from the European School Survey Project on Alcohol and Other Drugs (ESPAD) conducted in 2007 suggest that 43% of 15- to 16-year-old European students engaged in heavy episodic drinking during the past 30 days, and at least 50% of students reported having tried alcohol before the age of 13 [13]. Recent data on alcohol prevalence rates among US youth aged 12 to 20 years indicated that about 17.4% engaged in binge drinking in the past month and 5.5% were heavy drinkers [14].

Moreover, several meta-analyses of the efficacy of alcohol prevention programs indicate that the effects of current alcohol preventive approaches are fairly small [15-17]. To enhance the efficacy of alcohol prevention programs, new and advanced strategies are warranted. A promising endeavor may be the application of electronic media to deliver alcohol preventive materials. This delivery mode presents an opportunity to widely disseminate interventions in an easy and cost-effective way [18,19]. Moreover, since the majority of adolescents in Western countries have access to the Internet [20] and make frequent use of Internet technologies [21,22], Web-based interventions may be particularly suitable to target adolescent audiences.

Over the past years, Web-based delivery modes have successfully been applied to administer brief alcohol interventions [23-28]. These types of interventions can generally be described as short-term preventive consultations to detect problematic alcohol use in an early stage and to motivate nontreatment-seeking heavy drinkers to change their behavior or seek treatment. A core element of brief alcohol interventions is the presentation of discrepancies between what the client reports and what the client wants or what would be beneficial for him or her. The purpose is to increase motivation to change or modify his or her behavior. Therefore, most brief alcohol interventions consist of a screening procedure followed by personalized feedback that participants receive based on their answers to the screener questions. According to the literature, this tailored approach might be more effective than the delivery of a more general prevention message due to the fact that the receiver of the intervention is more likely to identify with personally relevant information and pay more attention to person-related information than to general information [29].

Initially, brief alcohol interventions were delivered in health care settings during face-to-face contact with a health professional [30]. More recently, other modes of delivery have been employed such as postal mail methods [31] and electronic methods via computer programs [32] as well as the Internet [23]. The latter Web-based approach may be more beneficial than the more traditional delivery methods because it allows easy access to large audiences and gives participants the opportunity to access the intervention at their own convenience, which may enhance participants’ feelings of privacy and anonymity. Furthermore, the inclusion of tailored information can be accomplished in an easier and more cost-effective way [19].

Previous trials suggest that Web-based brief alcohol interventions can reduce drinking in nonclinical adult populations, that is, problem drinkers [23], drinkers in the workplace [33], and heavy drinking [25,28,34] or mandated college students [24]. As indicated by these previous findings, Web-based brief alcohol interventions were effective in the short-term and midterm by reducing adults’ and young adults’ drinking rates at approximately 1 to 12 months after the intervention. Besides the preventive impact on adult and young adult drinkers, Web-based interventions may also hold potential for alcohol prevention among adolescents. Further insight into this topic is of great importance since early intervention in adolescents’ drinking careers might reduce the risk of escalation to more problematic drinking patterns. A short, personalized, Web-based intervention may specifically appeal to adolescent drinkers and may, therefore, effectively motivate them to modify their alcohol consumption. In spite of the potential benefits of Web-based brief alcohol interventions to target adolescent drinkers, previous studies have not addressed this topic to this date.

To our knowledge, the present study is the first to test the short-term effectiveness of a Web-based brief alcohol intervention among a sample of adolescents and young adults aged 15 to 20 years. Our objective was to additionally test the contribution of normative feedback to the effectiveness of this Web-based brief alcohol intervention. Normative feedback involves the presentation of comparative information about personal drinking levels and drinking levels of a relevant comparison group, such as same-aged peers. This prevention strategy was developed in response to a comprehensive body of literature showing that college students tend to overestimate their peers’ drinking levels [35]. According to the social norms approach, correction of these overstated perceptions about peers’ alcohol consumption levels will have an impact on youngsters’ alcohol use [36]. Although the effectiveness of providing normative feedback on peer alcohol use has been demonstrated in research on college samples [37], it is not clear whether this strategy would reduce alcohol use in adolescents. Moreover, it is possible that normative feedback might generate stronger effects in males than in females [36] due to the degree of specificity of the provided normative feedback [38] or due to differences in the flexibility of normative perceptions between girls and boys [39]. Therefore, a Web-based brief alcohol intervention could have a differential effect on drinking patterns in females and males in this age group. Notably, current interventions show some diversity in the type of normative information that is presented, which may affect the effectiveness of this component [36]. These differences exist mainly in the choice of reference group, which can vary in degree of specificity, that is, peers in general versus first year graduate students or same-sex peers. There is some evidence suggesting that prevalence information about specific reference groups has a stronger impact on college students’ normative perceptions and personal drinking behavior than does more general prevalence information [36]. In keeping with these preliminary findings, we aimed to test the impact of age- and gender-specific normative feedback on alcohol consumption levels in 15- to 20-year-old youths. In sum, the objective of the present study was to test the following research questions: (1) Is a Web-based brief alcohol intervention effective in reducing weekly drinking and encouraging moderate alcohol use in 15- to 20-year old youths? (2) Does inclusion of normative feedback contribute to the effectiveness of this Web-based brief alcohol intervention? (3) Does the impact of a Web-based brief alcohol intervention differ between males and females in this age group?

## Methods

### Study Design

The present study was a 3-arm randomized controlled trial in which participants screened as binge drinkers received 1 of 2 Web-based brief alcohol interventions or were assigned to the control group.

### Participants and Procedure

Volunteer members of an online access panel between 15 and 20 years of age were invited to complete an online survey on lifestyle and health behavior. We informed all participants that the assignment would occur by chance and that some participants did not need to evaluate an intervention but just had to answer some questions.

This online panel was set up and maintained by Flycatcher, a full service research agency affiliated with Maastricht University, the Netherlands. This agency is registered by the Dutch Data Protection Authority (number 1007001) and follows the European Society for Opinion and Marketing Research (ESOMAR) privacy policy stated by the European branch organization of research agencies. The research agency uses a double “opt in” procedure, which means that potential participants first indicate on a website that they would like to become a panel member by providing their email address. These potential panel members receive an email with information and a link to confirm or reject their membership. This double opt in procedure is regarded as a form of informed consent. Registered panel members can receive invitations by email with study information and a possibility to reject or confirm study participation. According to the Dutch and European research guidelines, at age 15 adolescents may be included in research if they grant their personal informed consent; parental consent is not required for this age group.

A total sample of 1012 participants was included and received an online questionnaire at baseline that contained items on demographic characteristics and alcohol use. Of these adolescents, 575 fulfilled the following inclusion criteria: 15 to 16 year old youths engaging in binge drinking at least once a month, or 17 to 20 year old youths engaging in binge drinking at least once a week. For females, binge drinking was defined as drinking more than 4 alcoholic consumptions per occasion and for males, more than 6 [12]. All included participants were randomly assigned to 1 of the following conditions: (1) Web-based brief alcohol intervention without normative feedback, (2) Web-based brief alcohol intervention with normative feedback, or (3) a control group. At 1 month after baseline, all participants received a short questionnaire with questions similar to those on the pretest. In addition, the 2 experimental groups received an email with a link to their assigned Web-based brief alcohol intervention. To assess the effectiveness of the 2 interventions, we conducted 2 posttests at 1 month and 3 months after the delivery of the interventions. At these posttests, both intervention groups and the control group received online questionnaires with questions about alcohol use. After each measurement or intervention, participants were sent 1 reminder after 4 days informing them that they had 1 week to complete the intervention or questionnaire. After completion, participants received vouchers to buy gifts, books, or movie tickets. No ethical approval was sought for this study.

### Randomization

An independent research agency assigned participants randomly to the conditions. The randomization was generated using a randomization function in Excel. Recruitment was stratified by sex, age, and educational level to obtain equal groups.

### Intervention

#### Description of the Web-based Interventions

The Web-based brief alcohol interventions consisted of 2 parts: (1) a questionnaire including items addressing participants’ drinking patterns, drinking motives, and health risk status and (2) personalized feedback based on participants’ answers to the earlier posed questions on the questionnaire including advice about moderate drinking. The advice for young adults aged 18 to 20 years was in line with the guidelines of the Dutch National Health Council recommending that men should not drink more than 2 drinks of alcohol per day and women, 1 drink of alcohol per day [40]. Adolescents under the age of 16 years received advice to abstain from alcohol. Adolescents aged 16 to 17 years were advised to abstain from alcohol, and if they drank alcohol, they were advised to drink moderately (not more than 1 or 2 drinks per occasion). The time to complete the intervention, which included filling out the screener questions and reading the personalized feedback, was estimated at 15 minutes.

#### Topics of the Interventions

The feedback was tailored to participant’s age (under 16, 16 to 17 years of age, and 18 years of age and older) and gender and organized along 4 topics for the intervention without normative feedback group, and 5 topics for the intervention with normative feedback group. These topics are described below.

##### Personal Drinking Behavior and Related Health Risks

Participants received a summary of the quantity and frequency of their drinking behavior. If participants’ alcohol use exceeded moderate drinking limits, they received information about how this could affect their health.

##### Drinking Motives and Suggestions to Reduce Alcohol Use

Drinking motives and suggestions to reduce alcohol use were instigated by risk-conducive motives, such as drinking to forget problems or to conform to peer pressure [41,42]

##### Risk of Developing Problematic Alcohol Use or Alcohol Dependence

Participants who showed increased risk due to specific physical reactions in response to alcohol [43], symptoms of physical dependence, or problematic alcohol use [44] were informed about their risk status and received suggestions to moderate their drinking and directions to seek further help.

##### Personal Perceptions About Own Alcohol Use and Related Risks

A summary of participants’ objective personal health risks was presented and set against their self-reported personal risk perceptions and motivation to engage in moderate drinking

##### Normative Feedback

The version of the drink test with normative feedback additionally provided an overview of how much participants thought their age mates would drink, how much their age mates actually drank, and how much the participants drank themselves. This information was presented in a bar chart showing each participant’s own weekly alcohol use, the actual prevalence rates of Dutch adolescents’ weekly alcohol use matched according to the participant’s sex and age, and the prevalence rates of Dutch adolescents’ weekly alcohol use as estimated by participants. The data on peers’ actual alcohol consumption levels were retrieved from alcohol prevalence estimates among same-age groups found in a nationally representative sample of high school students (included in feedback for adolescents aged 15 to 17 years) and the general population (included in feedback for young adults aged 18 to 20 years) [12,45]. Only participants who over estimated their peers’ alcohol consumption received prevalence rates pertaining to their peers’ actual alcohol use. If estimations were correct or lower than the actual prevalence rates, participants were informed that they had provided the correct estimation.

### Outcome Measures

Weekly alcohol consumption was assessed using the Dutch version of the Alcohol Weekly Recall [46]. Participants were asked to indicate retrospectively for the past 7 days, how many standard units they had consumed. For example: “Yesterday it was … (fill out the name of the day) and I consumed … standard units.” To ensure standardized responses, we provided for various beverages an overview of standard units.

The measure for moderate drinking was based on the item for weekly alcohol consumption, which was recoded as 0 = “no moderate drinking” and 1 = “moderate drinking.” Participants aged 15 to 17 years were labeled as “moderate drinkers” if they consumed no alcohol in the past week. Males aged 18 to 20 years were regarded as moderate drinkers if they consumed less than 14 alcoholic drinks in the past week, and same-aged females were regarded as moderate drinkers if they consumed less than 7 alcoholic drinks in the past week.

### Strategy of Analyses

Possible differences between the 3 conditions at baseline were tested using chi-square tests for sex, educational level, and moderate drinking and analyses of variance (ANOVA) for age and number of drinks a week, where 1 standard drink is equivalent to 10 grams of pure alcohol. The number of drinks per week (mean 14.91, SD 13.23) had a skewed distribution and a high level of kurtosis (skewness 1.86, kurtosis 6.40). We applied a log transformation on the number of drinks plus 1. The mean (SD) of the transformed variable was 2.37 (SD) 1.01, skewness became -0.78, and kurtosis was reduced to 0.14.

The randomized sample consisted of 575 respondents. However, 297 participants did not respond during the intervention period, which resulted in a sample of 278 participants who adhered to the intervention. We compared this sample with the dropout group with respect to sex, age, educational level, and alcohol use in the past week by conducting a logistic regression analysis and including group membership (0 = completers and 1 = dropouts) as the dependent variable. No differences were found for sex (odds ratio [OR] = 0.91, *P =* .58, 95% confidence interval [CI] 0.63 - 1.29) or weekly alcohol use (OR =1.10, *P* = .28, 95% CI 0.92 - 1.31). However, the samples differed on age (OR = 0.89, *P =* .05, 95% CI 0.79 - 0.99) and educational level (OR .66, *P =* .001, 95% CI 0.53 - 0.82) caused by a higher dropout rate among the younger participants and those with lower education levels.

The effects of the brief alcohol interventions (with or without normative feedback) on (the log transformation of) weekly alcohol consumption were tested with linear regression analyses. The intervention effects on moderate drinking were tested with logistic regression analyses. The intervention without normative feedback was represented by a dummy variable with 0 = control group and intervention group with normative feedback and 1 = group without normative feedback. The intervention with normative feedback was represented by a dummy variable with 0 = control group and intervention group without normative feedback and 1 = group with normative feedback. We also examined whether the intervention effects differed according to participant’s sex by including interaction terms of sex with both dummy variables (females = 0 and males =1). Participant’s age and educational level were included as covariates in all analyses. For the interpretation of interaction effects we used the SPSS macro MODPROBE [47]. This procedure allows the probing of interaction effects while controlling for other covariates and provides the coefficients of the conditional effects for each level of the moderator. Unfortunately, it was not possible to apply this analytic strategy to multiple imputed data. Therefore, we only conducted post hoc analyses for the completers-only sample.

To compare the control group with each of the 2 experimental groups, intention to treat (ITT) analysis is an adequate strategy for randomized controlled trials and is interpreted as including all respondents belonging to the original randomized groups [48]. In our case, we had high levels of dropout. Of the 575 randomized respondents, only 278 respondents completed both posttests (at the 1-month and 3-month follow-ups). These figures suggest a dropout rate of 51.6%, in terms of not filling in questionnaires. In our ITT analysis, we used the multiple imputation procedure of PASW Statistics 18 to deal with these missing values. We applied this procedure to the baseline sample of participants that fulfilled the criteria for inclusion in our study (n = 575). In addition, we analyzed the data for participants who completed both posttests at 1 month and 3 months, that is, the 278 participants in the completers-only group.

## Results

### Participants Flow

**Figure 1:**
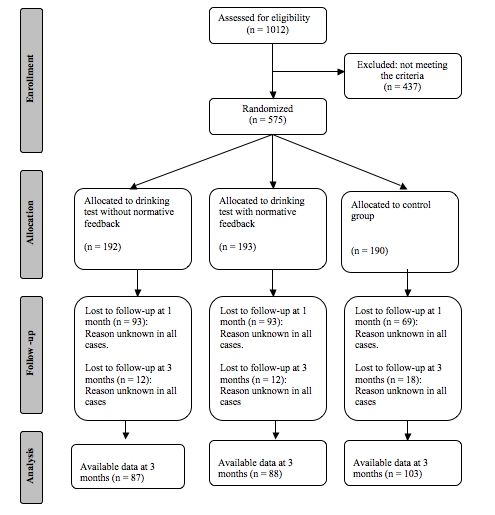
Trial schema

The flow of the participants through the trial is illustrated in Figure 1. Of the 1012 participants who completed the baseline, 575 participants fulfilled the inclusion criteria and were randomly assigned to 1 of 3 conditions. Of these potential participants, only 320 responded. At follow-up, 1 and 3 months after the intervention period, 278 completed both posttest measurements.

**Table 1:** Differences in demographic characteristics and alcohol consumption patterns among participants at baseline (n = 575)

Variable		NNF^a^ (n = 192)	NF^b^ (n = 193)	Control Group (n = 190)	Test Result	*P*
**Sex**					χ^2^_2_ 1.57	.46
	Males, n (%)	74 (38.5)	82 (42.5)	69 (36.3)		
	Females, n (%)	118 (61.5)	111 (57.5)	121 (63.7)		
Mean age (SD) in years		18.16 (1.55)	18.05 (1.54)	18.11 (1.59)	F_2,572_ = 0.26	.77
**Educational level**					χ^2^_4_ = 1.95	.75
	Low, n (%)	54 (28.1)	47 (24.4)	49 (25.8)		
	Medium, n (%)	72 (37.5)	71 (36.8)	66 (34.7)			
	High, n (%)	66 (34.4)	75 (38.8)	75 (38.5)		
N drinks of alcohol in past week, mean (SD)		14.2 (12.2)	15.1 (13.4)	14.8 (13.7)	F_2,572_ = 0.23	.80
Moderate drinking, n (%)		106 (55.2)	96 (49.7)	97 (51.1)	χ^2^_2_ = 1.25	.53

^a^ NNF= Intervention without normative feedback

^b^ NF = Intervention with normative feedback

### Sample Characteristics

Participants’ demographic and clinical characteristics assessed at baseline are summarized in Table 1. No differences were found between the 3 conditions, indicating that the randomization was successful. Of the 575 participants, 225 (39.1%) were male. Mean age (SD) of the participants was 18.1 years (1.56). Most of the participants had intermediate or high education levels (150 or 26.1% had low levels, 209 or 36.3%, intermediate, and 216 or 37.6%, high) and almost all (553 or 96.3%) were born in the Netherlands. The majority of participants (496 or 86.2%) were students, 6.1% (35) had a job and 7.7% (44) combined their study with a job.

### Weekly Alcohol Consumption

At baseline, participants (n = 575) had consumed on average 14.7 drinks of alcohol in the past week (11.5 for females, 19.7 for males). Participants’ weekly drinking rates at the 2 posttests (n = 278) were 11.9 drinks (8.6 for females, 17.0 for males) at the 1-month follow-up and 13.1 (9.6 for females, 18.5 for males) at the 3-month follow-up.

We conducted linear regression analyses to test whether the Web-based brief alcohol interventions reduced participants’ weekly alcohol consumption at 1 and 3 months after the intervention. In these analyses, we additionally tested possible moderation effects of participant’s sex by including the interactions between intervention and sex in the third step. Results of the third step of the equations are presented in Table 2. We controlled for demographic characteristics, that is, sex, age, and education level by including these variables in step 1 of the equations. When inspecting results of the first step, we found that only age was related to participants’ weekly alcohol use, suggesting that the older the participants, the higher their weekly drinking levels.

To determine whether the interventions had an overall effect on participants’ weekly drinking rates, we inspected the findings of the second step (not presented in Tables). Our data for the completers-only sample (n = 278) did not show any main effects. However, the multiple imputed dataset (n = 575) indicated that both interventions reduced weekly drinking at the 1-month follow-up (intervention without normative feedback, beta = -.24, *P =* .05; intervention with normative feedback, beta = -.34, *P =* .008) and the intervention without normative feedback reduced weekly drinking at the 3-month follow-up (beta = -.25, *P =* .04).

According to further analyses presented in Table 2, results of the third step showed significant interaction effects between sex and the Web-based brief alcohol intervention without normative feedback on participants’ weekly alcohol use at the 1-month follow-up (completers only, beta = -.80, *P =* .01; multiple imputed, beta = -.69, *P =* .009) and at the 3-month follow-up (completers only, beta = -.58, *P =* .05; multiple imputed, beta = -.75, *P =* .004). These findings suggest that the impact of the Web-based brief alcohol intervention without normative feedback on weekly alcohol use at the 1- and 3- month follow-up differed between males and females. For the further interpretation of these interactions, we used the SPSS Macro MODPROBE and calculated the coefficients of the focal predictor (Web-based brief alcohol intervention without normative feedback) at both levels of the moderator (participant’s sex). These post hoc analyses suggested that males who received the intervention without normative feedback were more likely to reduce their weekly alcohol use at the 1-month (beta = -.43, *P =* .08) and 3-month follow-up (beta = -.45, *P =* .049). In contrast, females who received the intervention without normative feedback were more likely to increase their weekly alcohol use at the 1-month follow-up (beta = .37, *P =* .06). If we express these estimates in number of drinks, results would imply that the intervention without normative feedback reduced the weekly drinking rate at the 1-month follow-up in males by 5 drinks of alcohol (from 12.8 to 7.8 drinks) and increased the weekly drinking rate in females by 1.6 drinks (from 3.8 to 5.4 drinks). The estimated effect of the intervention without normative feedback on males’ weekly alcohol use at the 3-month follow-up amounted to a reduction of 5.3 drinks (from 13.5 to 8.2 drinks).

Regarding the intervention with normative feedback, our results showed interaction effects with participant’s sex on weekly alcohol use at the 1-month follow-up for the completers-only group (beta = -.74, *P =* .02) and at both time points for the multiple imputed sample (at 1 month, beta = -.64, *P =* .01, and at 3 months, beta = -.65, *P =* .01) suggesting that the impact of the intervention differed between males and females. Post hoc analyses showed that males who received the intervention with normative feedback were more likely to reduce their weekly alcohol use at 1 month (completers only [n = 278], beta = -.49, *P =* .03). This implies that the intervention with normative feedback reduced weekly alcohol use in males by 5.6 drinks (from 13.5 to 7.9). Unfortunately, it was not possible to test the interaction effect on weekly drinking at the 3-month follow-up in the multiple imputed sample.

**Table 2:** Multiple regression analyses predicting weekly alcohol consumption at the 1-month follow-up and the 3-month follow-up

	Weekly Alcohol Consumption at the 1-Month Follow-up						Weekly Alcohol Consumption at the 3-Month Follow-up					
	Completers Only n = 278			Multiple Imputed n = 575			Completers Only n = 278			Multiple Imputed n = 575		
	Beta	SE	*P*	Beta	SE	*P*	Beta	SE	*P*	Beta	SE	*P*
Sex (males)	1.16	.21	< .001	0.80	.18	< .001	1.01	.20	< .001	0.86	.11	< .001
Age	0.15	.04	< .001	0.13	.03	< .001	0.21	.04	< .001	0.15	.03	< .001
Education	0.07	.08	.37	0.23	.07	< .001	0.29	.08	< .001	0.32	.06	< .001
NNF^a^	0.37	.20	.06	0.01	.16	.95	0.13	.18	.49	0.02	.16	.89
NF^b^	0.25	.20	.21	-0.09	.16	.56	0.18	.19	.34	0.03	.16	.83
Interaction NNF by sex	-0.80	.31	.01	-0.69	.26	.009	-0.58	.30	.05	-0.75	.26	.004
Interaction NF by sex	-0.74	.31	.02	-0.64	.26	.01	-0.34	.29	.25	-0.65	.25	.01

^a^ NNF= Intervention without normative feedback

^b^ NF = Intervention with normative feedback

### Moderate Drinking

Tables 3 and 4 present findings about the effect of the Web-based brief alcohol interventions on participants’ levels of moderate drinking at the 1- and 3-month follow-up. In our analyses, we controlled for participant’s sex, age (continuous variable), and education level (continuous variable). Although the Tables only present the coefficients of the third step, we also inspected the main effects of participants’ demographic characteristics and of the intervention in the earlier 2 steps. Results indicated that older participants were more likely to show moderate drinking at the 1-month follow-up and the 3-month follow-up. In addition, our data showed a borderline significant effect of sex on moderate drinking at the 3-month follow-up, suggesting that females were more likely to engage in moderate drinking than males.

Further, analyses based on the multiple imputed sample (n=575) indicated a borderline significant main effect of the Web-based brief alcohol intervention without normative feedback on responsible drinking at the 3-month follow-up suggesting that participants who received the Web-based brief alcohol intervention were slightly more likely to engage in responsible drinking 3 months after the intervention (beta = .40, *P =* .07).

Our findings further showed a significant interaction between sex and Web-based brief alcohol intervention without normative feedback at the 1-month follow-up for the completers-only group and a borderline significant interaction for the multiple imputed sample (completers only, OR = 3.76, *P =* .04, 95% CI 1.05 - 13.49; multiple imputed sample [n = 575], OR = 3.00, *P =* .08, 95% CI .89 - 10.12). At the 3-month follow-up, we found significant interactions between the Web-based brief alcohol intervention without normative feedback and participant’s sex for both samples (completers only, OR = 4.34, *P =* .04, 95% CI 1.18 - 15.95; multiple imputed sample, OR = 3.65, *P =* .006, 95% CI 1.44 - 9.25).

Post hoc probing of these interactions for the completers-only group demonstrated that males who received the intervention without normative feedback were more likely to engage in moderate drinking but only at the 3-months follow-up (beta = 1.21, *P =* .02). In contrast, data for females showed that those who received the intervention without normative feedback were less likely to engage in moderate drinking at the 1-month follow-up (beta = -.82, *P =* .046). Post hoc analyses further demonstrated that males who received the intervention with normative feedback were slightly more likely to engage in moderate drinking at the 1-month follow-up (beta = .83, *P =* .09). At the 3-month follow-up, the effect of the intervention with normative feedback on responsible drinking differed for males and females, but closer inspection showed that there were no significant changes in the likelihood to engage in moderate drinking in males or females.

**Table 3:** Logistic regression analyses predicting moderate drinking at the 1-month follow-up (intention to treat analysis)

	Moderate Drinking at the 1-Month Follow-up					
	Completers Only n = 278			Multiple Imputed n = 575		
	OR	95% CI	*P*	OR	95% CI	*P*
Sex (males)	0.52	(0.22 - 1.21)	.13	0.61	(0.27 - 1.38)	.23
Age	1.40	(1.17 - 1.67)	< .001	1.37	(1.16 - 1.62)	< .001
Education	0.95	(0.68 - 1.32)	.75	0.97	(0.70 - 1.32)	.83
NNF ^a^	0.44	(0.19 - 0.99)	.05	0.53	(0.25 – 1.14)	.11
NF ^b^	0.74	(0.33 - 1.62)	.45	0.96	(0.46 – 1.99)	.92
Interaction NNF by sex	3.76	(1.05 - 13.49)	.04	3.00	(0.89 - 10.12)	.08
Interaction NF by sex	3.12	(0.90 - 10.76)	.07	2.22	(0.69 - 7.14)	.18

^a^ NNF = Intervention without normative feedback

^b^ NF = Intervention with normative feedback

**Table 4:** Logistic regression analyses predicting moderate drinking at the 3-month follow-up (intention to treat analysis)

Moderate Drinking at the 3-Month Follow-up						
	Completers Only n = 278			Multiple Imputed n = 575		
	OR	95% CI	*P*	OR	95% CI	*P*
Sex (males)	0.35	(0.14 - 0.89)	.03	0.40	(0.21 - 0.75)	.004
Age	1.16	(0.97 - 1.38)	.09	1.00	(0.89 - 1.13)	.96
Education	0.56	(0.40 - 0.79)	.001	0.55	(0.43 - 0.69)	<.001
NNF ^a^	0.77	(0.35 - 1.68)	.05	0.91	(0.52 - 1.61)	.75
NF ^b^	0.55	(0.24 - 1.26)	.45	0.77	(0.44 - 1.37)	.38
Interaction NNF by sex	4.34	(1.18 - 15.95)	.04	3.65	(1.44 - 9.25)	.006
Interaction NF by sex	3.10	(0.81 - 11.85)	.07	3.00	(1.23 – 7.27)	.01

^a^ NNF= Intervention without normative feedback

^b^ NF = Intervention with normative feedback

## Discussion

### Principal Results and Comparison With Prior Research

The purpose of the present study was to test the effectiveness of a Web-based brief alcohol intervention in reducing weekly alcohol use and promoting moderate drinking among 15- to 20-year-old drinkers and to determine whether inclusion of normative feedback would increase its effectiveness. The study findings showed some main intervention effects, but these were primarily found for weekly drinking and only in the multiple imputed data of the original sample. Moreover, at the 3-months follow-up, the inclusion of normative feedback did not contribute to the effectiveness of the brief alcohol intervention since only the intervention without normative feedback resulted in a decrease in participants’ weekly drinking rates, 3 months after exposure to the intervention.

Additional analyses for both datasets suggested that the impact of the Web-based brief alcohol interventions differed for males and females. According to findings from both datasets, males who received the Web-based brief alcohol intervention showed lower levels of weekly alcohol use and were more likely to have engaged in moderate drinking at the 1- and 3-month follow-up. The estimated effect of the intervention without normative feedback on males’ weekly drinking rates amounted to a reduction of 5 drinks of alcohol at the 1-month follow-up and 5.2 drinks at the 3-month follow-up. For females, the intervention did not yield any effects, except that we found a small unfavorable effect of the intervention without normative feedback 1 month after the intervention. More specifically, females who received the intervention without normative feedback were less likely to engage in moderate drinking and showed an estimated increase in weekly drinking of 1.6 drinks of alcohol at the 1-month follow-up. In addition, our data indicated that the brief alcohol intervention with normative feedback increased responsible drinking in male drinkers but only at the 1-month and not at the 3-month follow-up. The estimated effect of the Web-based brief alcohol intervention with normative feedback on males’ weekly drinking levels at the 1-month-follow up amounted to a reduction of 5.6 drinks of alcohol.

Our finding that a Web-based brief alcohol intervention increased moderate drinking in males aged 15 to 20 years is encouraging for the further implementation of brief alcohol interventions among late adolescents. However, it should be noted that we used a short follow-up of 3 months. Moreover, it is striking that our intervention reduced alcohol use predominantly in males and hardly in females. The latter finding contradicts outcomes from previous research showing either an overall effect of brief alcohol interventions [49,50] or an even stronger effect in women [37,51]. As shown by a review of the impact of alcohol abuse interventions on drinking in college samples, interventions were more effective in reducing alcohol-related problems if the sample contained more women [50]. However, the evidence for greater effectiveness of brief alcohol interventions in females compared to males stems from research on brief interventions that were delivered face-to-face or by postal mail, whereas our intervention was Web-based. A recent study demonstrated that female college students receiving a face-to-face intervention showed greater reductions in alcohol use than female students who received a computer intervention [52]. These findings suggest that female drinkers are less responsive to computer-tailored brief alcohol interventions. However, this explanation does not account for the contrasting evidence between the present study and earlier research showing the effectiveness of Web-based brief alcohol interventions in a total sample of both males and females [28]. Since it is not clear whether the sex-specific effect of our intervention was due to our younger sample or other factors, we recommend further research on this issue.

Surprisingly in our study, males who were exposed to the brief alcohol intervention with normative feedback showed decreases in weekly drinking but no increases in moderate drinking levels at the 3-month follow-up. In contrast, males who received the brief alcohol intervention without normative feedback showed decreased weekly drinking 1 month and 3 months after the intervention and higher levels of moderate drinking 3 months after the intervention. Thus, our data suggest that the inclusion of normative feedback does not contribute to the effectiveness of the tested brief alcohol intervention in adolescent male drinkers in the long term. This finding is not in line with previous research demonstrating the effectiveness of normative feedback, particularly for males [53]. Logically, the lower impact of the brief alcohol intervention with normative feedback cannot be explained by the fact that we used age-specific and sex-specific normative feedback, since previous findings suggest that information about specific reference groups would increase and not decrease the effectiveness of normative feedback. However, a possible explanation might be that we tested a younger sample than has been examined in previous research. To date, the effectiveness of normative feedback in reducing alcohol use in adolescent drinkers has not been addressed in previous research. It might be the case that the presentation of normative comparison information has fewer long-lasting effects in adolescent drinkers compared with college students. To gain further insight into this matter, more research is needed on the effectiveness of normative feedback in adolescent samples.

### Limitations

Several limitations need to be considered when discussing the implications of our study. First, it is important to note that our findings are based on a convenience sample with some inevitable dropout, particularly among the younger and less highly educated participants. Also, since we used a convenience sample of online panel members who were binge drinkers, it is not clear whether our findings would generalize to more clinical samples. Importantly, 51.6% of the panel members who agreed to participate in the research did not respond after being allocated to the intervention or control condition. Unfortunately, we do not have any information about what caused this high dropout rate. It is possible that a substantial portion of the recruited participants were not interested in the intervention. This is likely since participants were not selected on the basis of their own treatment motivations but were included if they met criteria for binge drinking. These high dropout rates suggest that a considerable part of the adolescent population might not use or benefit from Web-based brief alcohol interventions. This is an important issue for the further implementation of Web-based brief alcohol interventions and should be further investigated in future research. Second, we used self-reported data to assess participants’ alcohol consumption levels. This type of measurement may show response bias due to social desirability concerns and memory effects. However, a number of studies confirmed the validity of self-reports of alcohol use [54-57] suggesting that self-reports can be used to assess drinking behavior. Still, to further improve the assessment of participants’ drinking levels future studies could, for instance, include other types of measurements such as Ecological Momentary Assessment (EMA) or direct observations. Finally, the utility of the tested intervention should be further examined by testing its effectiveness in the longer term after repeated exposure and among light drinkers.

### Conclusion

Present findings suggest that a Web-based brief alcohol intervention can be effective in reducing weekly alcohol use and encouraging moderate drinking in 15- to 20-year-old males over a period of 1 to 3 months. Inclusion of normative feedback does not seem to enhance the effectiveness of the intervention in encouraging moderate drinking 3 months following the intervention. Moreover, the interventions do not seem effective in females in this age group, and the intervention without normative feedback even showed small unfavorable effects at the 1-month follow-up.

## Abbreviations

ANOVA: analyses of variance
EMA: Ecological Momentary Assessment
ESOMAR: European Society for Opinion and Marketing Research
ESPAD: European School Survey Project on Alcohol and Other Drugs
ITT: intention to treat
